# Supplementary material for: TRIM2 Selectively Regulates Inflammation-Driven Pathological Angiogenesis without Affecting Physiological Hypoxia-Mediated Angiogenesis
Source: Int J Mol Sci. 2024 Mar 15;25(6):3343. doi: 10.3390/ijms25063343 (PMC10970352; doi:10.3390/ijms25063343)
Supplement: Supplementary file 1 [file ijms-25-03343-s001.zip › ijms-2893460-supplementary.pdf]

SUPPLEMENTAL FIGURE S1

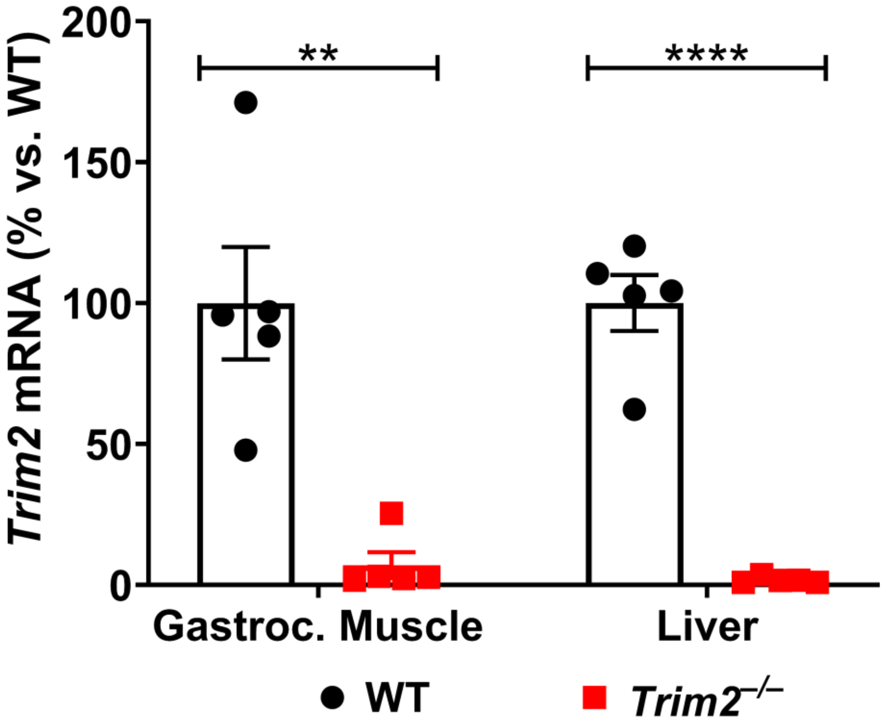

Supplemental Figure S1: Mouse *Trim2* mRNA levels were determined using qPCR from gastrocnemius muscle and liver tissues from wildtype (WT) and *Trim2*$^{-/-}$ mice (N = 5/group). Results are mean±SEM and normalized to WT mice. ** $p < 0.01$, **** $p < 0.0001$ by unpaired student's t-test.

SUPPLEMENTAL FIGURE S2

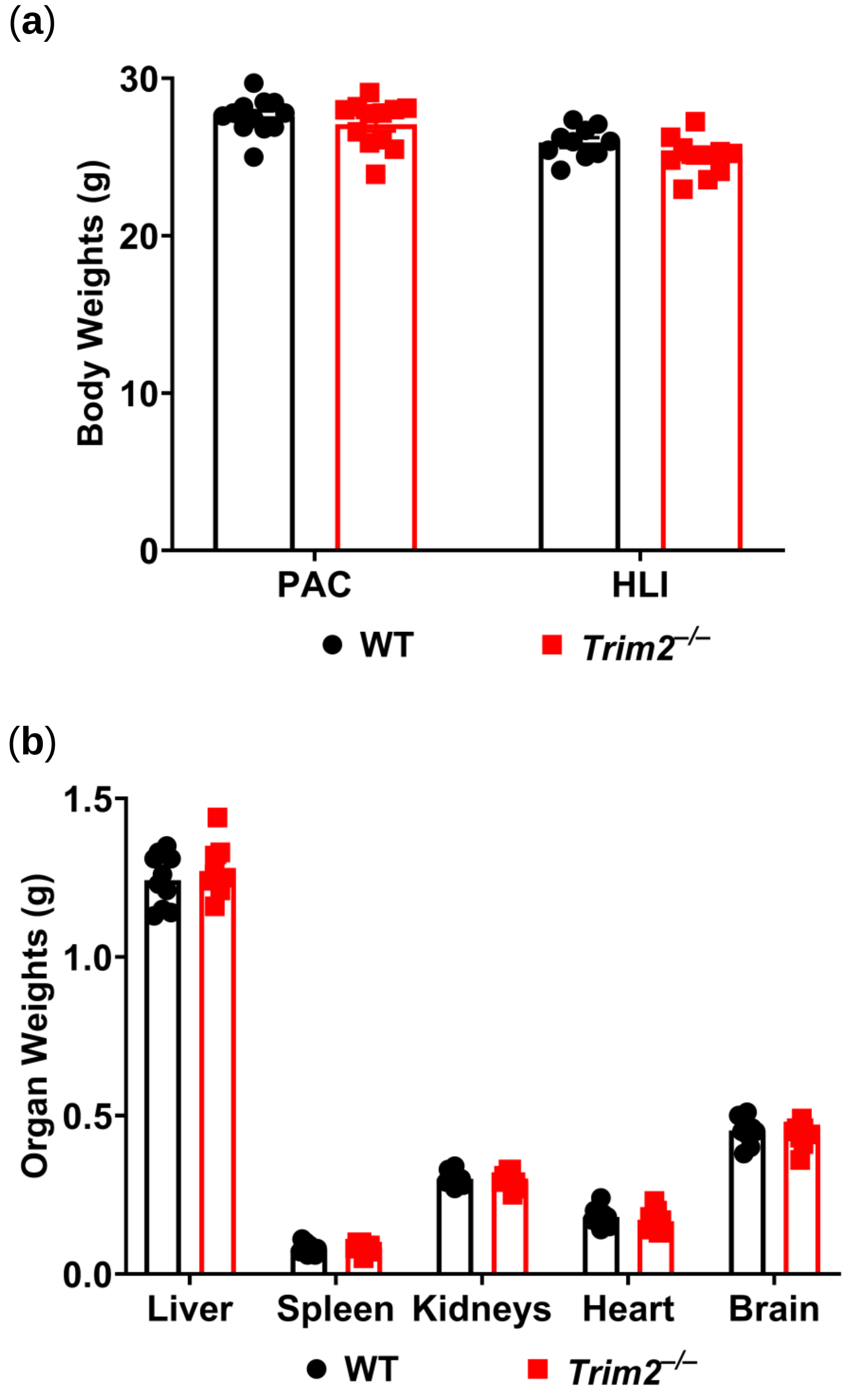

Supplemental Figure S2: (a) Total body weight of wildtype (WT) and *Trim2*$^{-/-}$ mice at the conclusion of either the periarterial cuff (PAC, *N*=12/group) or hindlimb ischemia (HLI, *N*=10–11/group) studies. **(b)** Weights of individual organs (*N*=10/group). Results are mean ± SEM and normalized to WT mice.

SUPPLEMENTAL FIGURE S3

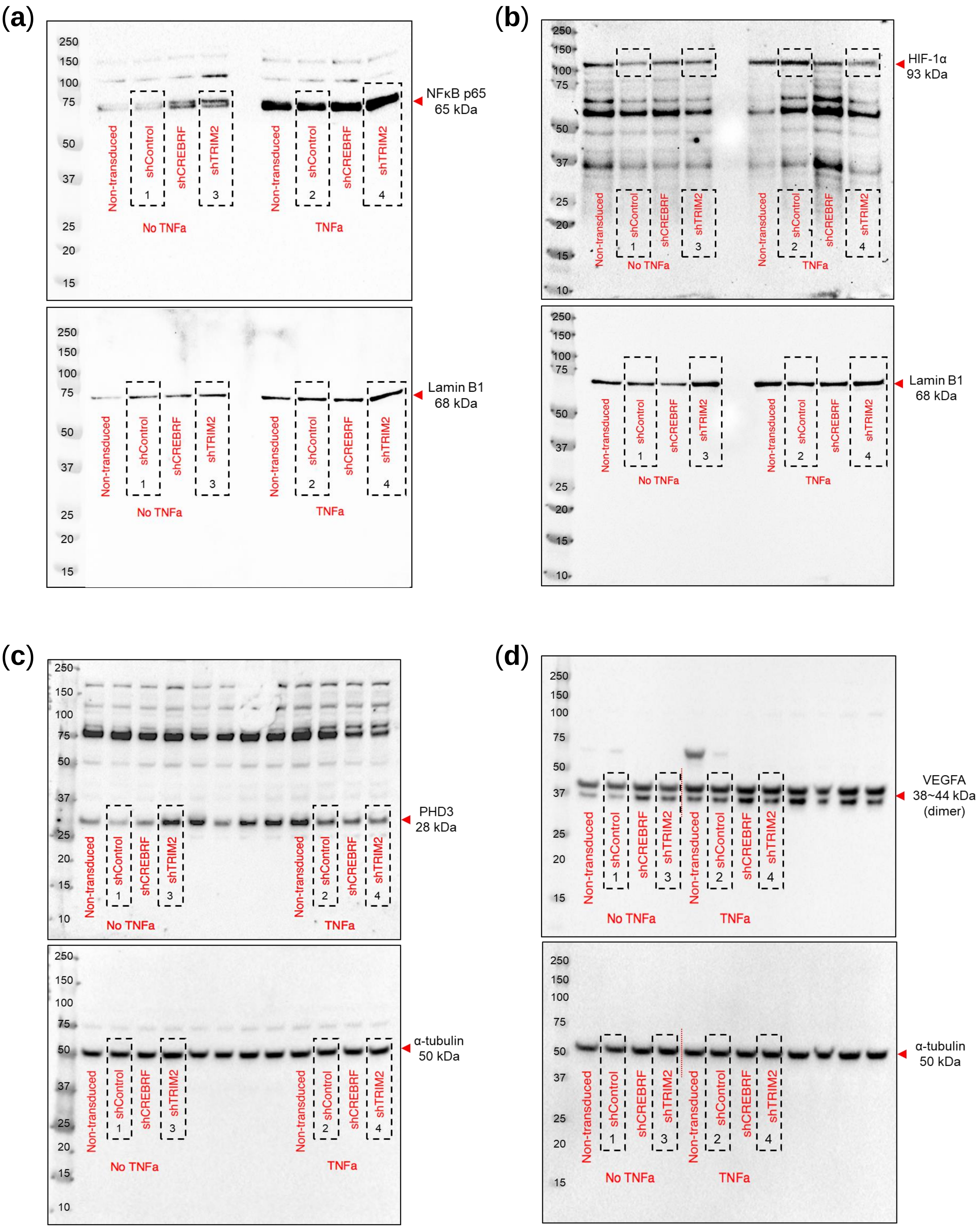

Supplemental Figure S3: Full blots of **(a)** NFκB p65 and Lamin B1 for Figure 3h; **(b)** HIF-1α and Lamin B1 for Figure 3i; **(c)** PHD3 and α-tubulin for Figure 3j; and **(d)** VEGFA and α-tubulin for Figure 3k. Dotted lines show the cropping location and the order in which the bands are presented.

SUPPLEMENTAL FIGURE S4

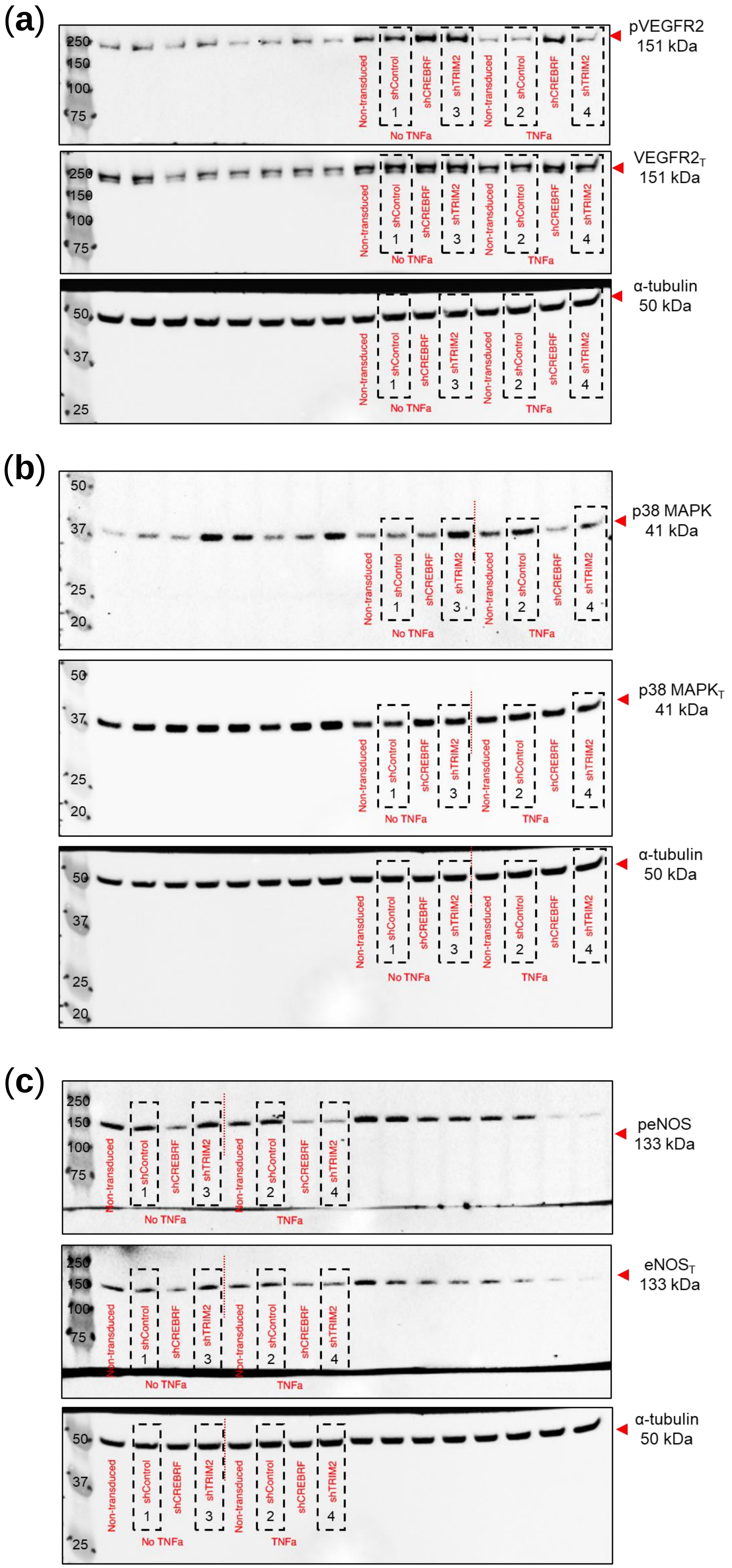

Supplemental Figure S4: Full blots of **(a)** phosphorylated (Tyr^{1175}) VEGFR2, total VEGFR2 and α-tubulin for Figure 3l; **(b)** phosphorylated (Thr^{180}/Tyr^{182}) p38 MAPK, total p38 MAPK and α-tubulin for Figure 3m; and **(c)** phosphorylated (Ser^{1177}) eNOS, total eNOS and α-tubulin for Figure 3n. Dotted lines show the cropping location and the order in which the bands are presented.

SUPPLEMENTAL FIGURE S5

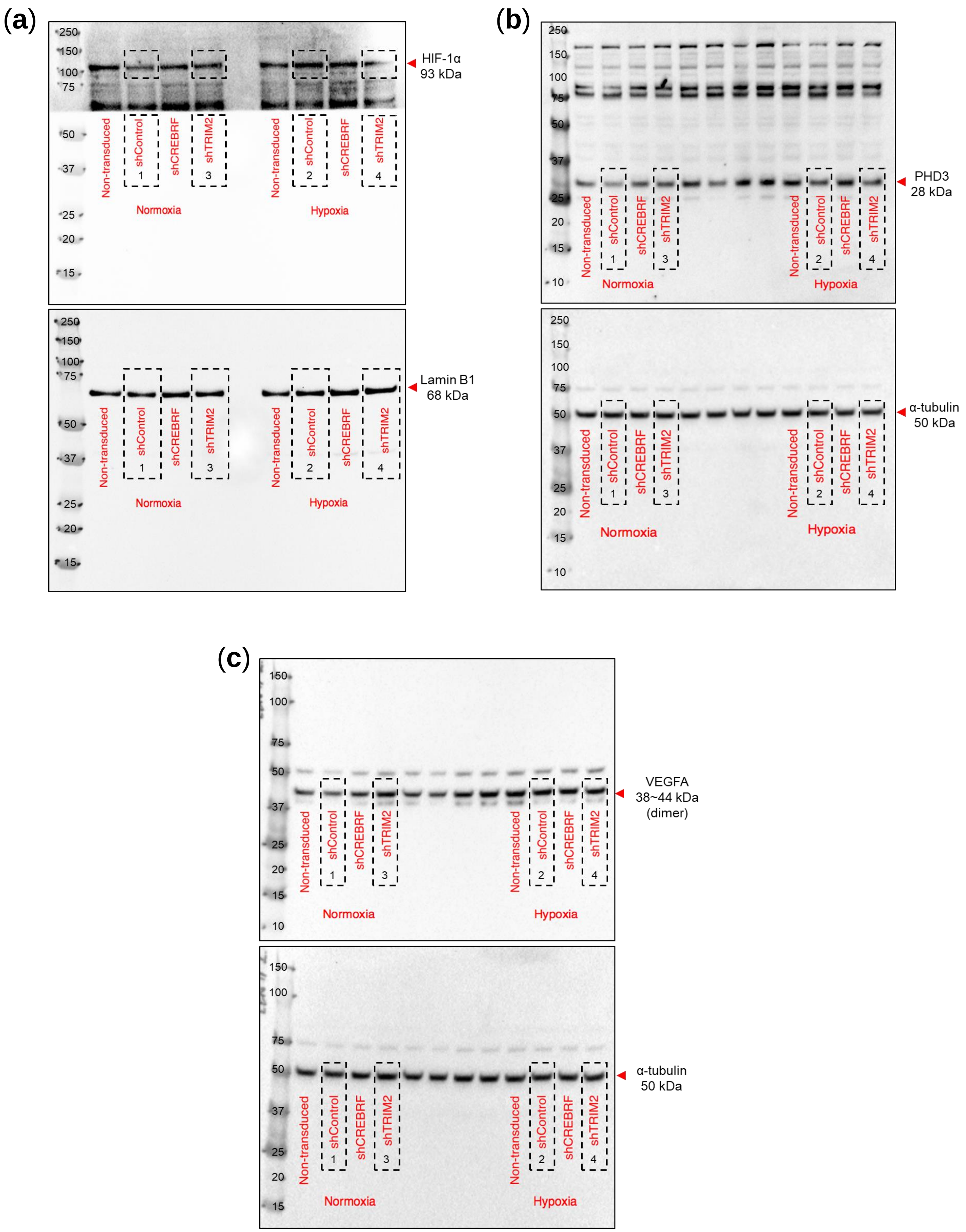

Supplemental Figure S5: Full blots of **(a)** HIF-1α and Lamin B1 for Figure 5g; **(b)** PHD3 and α-tubulin for Figure 5h; and **(c)** VEGFA and α-tubulin for Figure 5i. Dotted lines show the cropping location and the order in which the bands are presented.

SUPPLEMENTAL FIGURE S6

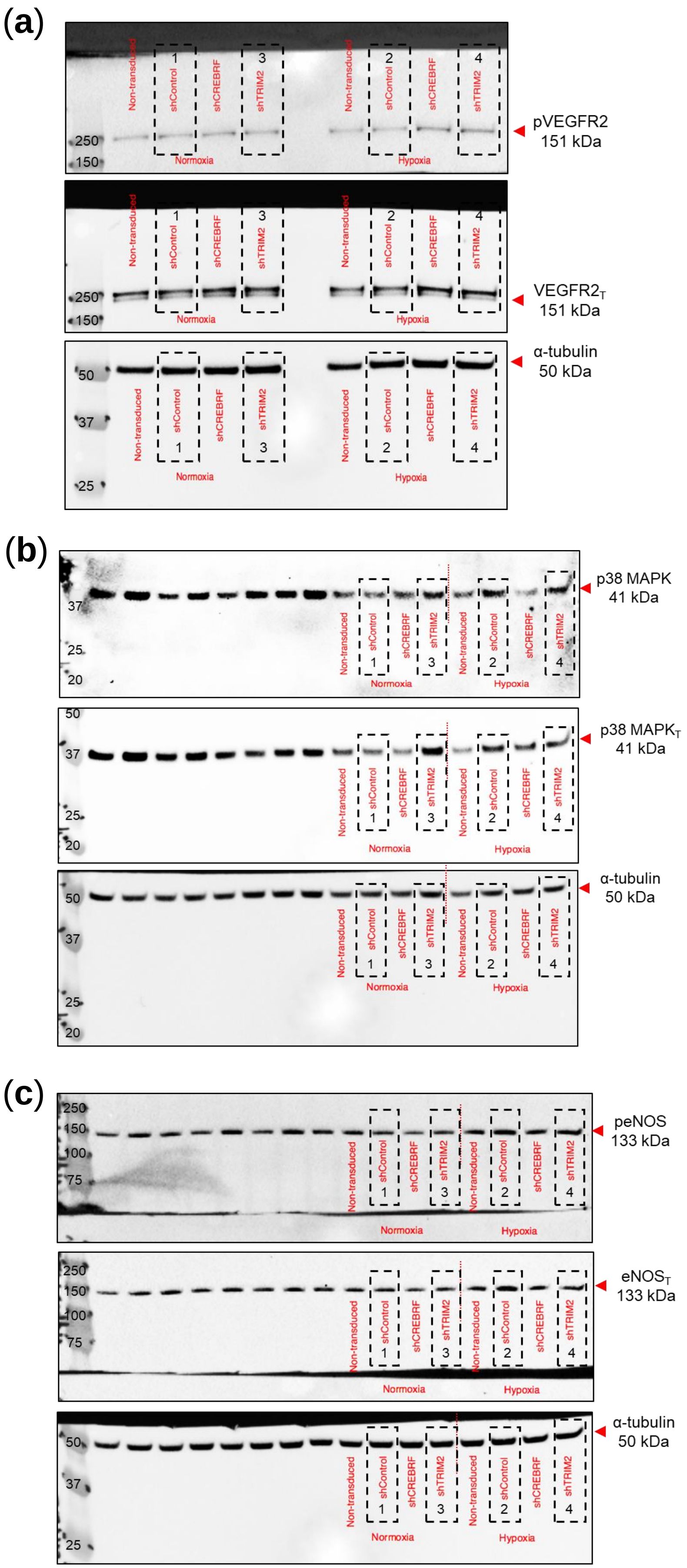

Supplemental Figure S6: Full blots of **(a)** phosphorylated (Tyr[1175]) VEGFR2, total VEGFR2 and α-tubulin for Figure 5j; **(b)** phosphorylated (Thr[180]/Tyr[182]) p38 MAPK, total p38 MAPK and α-tubulin for Figure 5k; and **(c)** phosphorylated (Ser[1177]) eNOS, total eNOS and α-tubulin for Figure 5l. Dotted lines show the cropping location and the order in which the bands are presented.
